# Abnormal septal convexity into the left ventricle occurs in subclinical hypertrophic cardiomyopathy

**DOI:** 10.1186/s12968-015-0160-y

**Published:** 2015-07-30

**Authors:** Patricia Reant, Gabriella Captur, Mariana Mirabel, Arthur Nasis, Daniel M. Sado, Viviana Maestrini, Silvia Castelletti, Charlotte Manisty, Anna S. Herrey, Petros Syrris, Maite Tome-Esteban, Sharon Jenkins, Perry M. Elliott, William J. McKenna, James C. Moon

**Affiliations:** Division of Cardiovascular Imaging and Inherited Cardiac Disease Unit, The Heart Hospital, Institute of Cardiovascular Science, University College London, London, UK; Hôpital Cardiologique du Haut-Levêque (Pessac), CHU de Bordeaux, Université de Bordeaux, Bordeaux, France; INSERM U970, Paris Cardiovascular Research Center PARCC, Paris, France

**Keywords:** Hypertrophic cardiomyopathy, Cardiovascular magnetic resonance, Septal curvature, Genetics

## Abstract

**Background:**

Sarcomeric gene mutations cause hypertrophic cardiomyopathy (HCM). In gene mutation carriers without left ventricular (LV) hypertrophy (G + LVH-), subclinical imaging biomarkers are recognized as predictors of overt HCM, consisting of anterior mitral valve leaflet elongation, myocardial crypts, hyperdynamic LV ejection fraction, and abnormal apical trabeculation. Reverse curvature of the interventricular septum (into the LV) is characteristic of overt HCM. We aimed to assess LV septal convexity in subclinical HCM.

**Methods:**

Cardiovascular magnetic resonance was performed on 36 G + LVH- individuals (31 ± 14 years, 33 % males) with a pathogenic sarcomere mutation, and 36 sex and age-matched healthy controls (33 ± 12 years, 33 % males). Septal convexity (SCx) was measured in the apical four chamber view perpendicular to a reference line connecting the mid-septal wall at tricuspid valve insertion level and the apical right ventricular insertion point.

**Results:**

Septal convexity was increased in G + LVH- compared to controls (maximal distance of endocardium to reference line: 5.0 ± 2.5 mm *vs*. 1.6 ± 2.4 mm, p ≤ 0.0001). Expected findings occurred in G + LVH- individuals: longer anterior mitral valve leaflet (23.5 ± 3.0 mm *vs*. 19.9 ± 3.1 mm, p ≤ 0.0001), higher relative wall thickness (0.31 ± 0.05 *vs*. 0.29 ± 0.04, p ≤ 0.05), higher LV ejection fraction (70.8 ± 4.3 % *vs*. 68.3 ± 4.4 %, p ≤ 0.05), and smaller LV end-systolic volume index (21.4 ± 4.4 ml/m^2^*vs*. 23.7 ± 5.8 ml/m^2^, p ≤ 0.05). Other morphologic measurements (LV angles, sphericity index, and eccentricity index) were not different between G + LVH- and controls.

**Conclusions:**

Septal convexity is an additional previously undescribed feature of subclinical HCM.

## Background

Hypertrophic cardiomyopathy (HCM) is the most common inherited monogenetic cardiac disease [1]. Approximately 60 % of cases are known to be caused by the presence of inherited sarcomeric protein mutations. However these mutations may have incomplete expression with a variable phenotype and age-related expression. The first degree relatives of individuals with HCM therefore have approximately a 50 % pretest probability of genetic carriage. Genotyping may help identify this but is sometimes inconclusive or non-contributory. There is however, a subtle subclinical phase of HCM. Gene mutation carriers without left ventricular (LV) hypertrophy (G + LVH-) have been found by cardiovascular magnetic resonance (CMR) to have: elongated anterior mitral valve leaflets (AMVL) [2], myocardial crypts [3–5], hyperdynamic LV ejection fraction, and abnormal apical LV trabeculation (measured using fractal analysis) [6, 7]. Additional features that may be present are markers of fibrosis [8, 9] and echocardiographic markers of diastolic dysfunction [10]. We suspect that this list is incomplete: specifically, that CMR research has really not fully exploited the insights from echocardiography and that some features are being missed particularly in term of LV global and regional morphology.

The collaboration between CMR specialists and echocardiologists permits the sharing of skills and ideas. For example, LV morphology has been extensively and classically characterized by echocardiography using specific parameters and indexes (sphericity, eccentricity, relative wall thickness) [11], most of which could also be readily applied to CMR [12].

Patients with overt HCM (with clinical hypertrophy) display abnormal LV septal curvature: in the normal heart the interventricular septum is either in a neutral position or has a characteristic convexity into the right ventricle (RV); whilst overt HCM is associated with reverse septal curvature leading to a typical “banana-like” LV cavity [13–18]. The presence of reverse septal curvature “convexity” in overt HCM is associated with a higher prevalence of identified sarcomere gene mutation [18] and may have an impact on regional strain in these patients [19]. Furthermore, various angles (angle between the LV long axis and mitral valve, angle between papillary muscles insertion points, and angle between LV long axis and aortic root) are different in overt HCM compared to those in a normal heart [20]. We therefore hypothesized that abnormal LV septal curvature, as well as several other morphological abnormalities (angles, indexes), may be present before hypertrophy in G + LVH- individuals and be a component of subclinical HCM.

## Methods

### Study population

A collaborative group at the Heart Hospital (University College London, United Kingdom) have previously created and published a case–control cohort [7] composed of G + LVH- HCM participants matched to healthy volunteers on the basis of age (±8 years), sex, body surface area (BSA ±10 %), and ethnicity. Inclusion criteria for the G + LVH− group included: (1) maximal LV wall thickness <13 mm by CMR and mass within the normal range relative to BSA, age, and sex; (2) sinus rhythm, no LVH, and no pathological Q waves/T-wave inversion on 12-lead electrocardiography; and (3) no causes of secondary LVH (valve disease, hypertension). Healthy volunteers had no history of cardiovascular disease or hypertension, a normal physical examination, no family history of inheritable cardiomyopathy or sudden cardiac death, and no personal history of cardiac symptoms or cardiovascular disease (including hypertension) and with a normal physical examination and ECG. Exclusion criteria for all participants were the presence of conventional contraindications for CMR, claustrophobia, and arrhythmias (e.g., atrial fibrillation, frequent atrial or ventricular ectopics). An ethics committee of the UK National Research Ethics Service approved the generic analysis of anonymized clinical scans. The genotyping project was approved by the UCL/UCLH Joint Research Ethics Committee. All participants gave written informed consent conforming to the Declaration of Helsinki (fifth revision, 2000). Study data were collected and managed using REDCap electronic data capture tool (Research Electronic Data Capture, REDCap Software - Version 5.9.6, http://www.project-redcap.org/).

### Electrocardiography

Standard 12-lead electrocardiography was performed in the supine position during quiet respiration. LVH was evaluated with the Romhilt-Estes [21] and electrocardiographic European criteria [22, 23]. Electrocardiographic images were analyzed by an experienced observer blinded to clinical and CMR data.

### Genetic screening

Genomic analysis of this cohort has been previously described in detail [7, 24, 25]. G+ individuals were defined as the ones carrying a known disease causing mutation in one of the following sarcomere genes: myosin-binding protein C (MYBPC3), β-myosin heavy chain (MYH7), troponin T (TNNT2), troponin I (TNNI3), myosin regulatory light chain (MYL2), myosin essential light chain (MYL3), tropomyosin (TPM1), and cardiac α-actin (ACTC1).

### Cardiovascular magnetic resonance image acquisition

Standard clinical scans (localizers, 3 long-axis views, black and white blood images, full LV short-axis stack) were performed using a 1.5-T magnet (Avanto, Siemens Medical Solutions®, Erlangen, Germany). CMR short-axis volumetric studies [26] were acquired from retrospectively gated, breath-held, balanced, steady-state free-precession cines (slice thickness, 7 mm; interslice gap, 3 mm; flip angle, 60°–80°; repetition time, 3.0 ms; echo time, 1.33 ms; field of view read typically, 380 mm; phase resolution, 75 %; typical acquired voxel size, 1.5 × 1.7 mm; 12 lines per segment). Late gadolinium enhancement images acquired through an inversion recovery turbo fast low-angle shot sequence were obtained 7 to 15 min after injection of 0.1 mmol/kg gadolinium-diethylenetriamine penta-acetic acid.

### Cardiovascular magnetic resonance analysis

The morphology, systolic function and the structure of the LV were evaluated by cardiologists experienced in CMR (PR, MM). All CMR measurements were blinded to gene status. The presence of fibrosis and the structure of the LV were evaluated by other cardiologists experienced in CMR (JCM, GC, DMS).

### Standard CMR measurements

LV volumes, LV ejection fraction (EF), LV outflow tract diameter, and LV mass were determined according to standardized CMR methods [27] (papillary muscles were included in the LV mass). LV wall thickness was measured at the septum and posterior wall on end-diastolic short-axis cine frames. The ratio of maximal septal diastolic wall thickness to posterior wall thickness was calculated, as well as relative wall thickness according to echocardiographic guidelines [11].

### Measurement of septal convexity

**Fig. 1:**
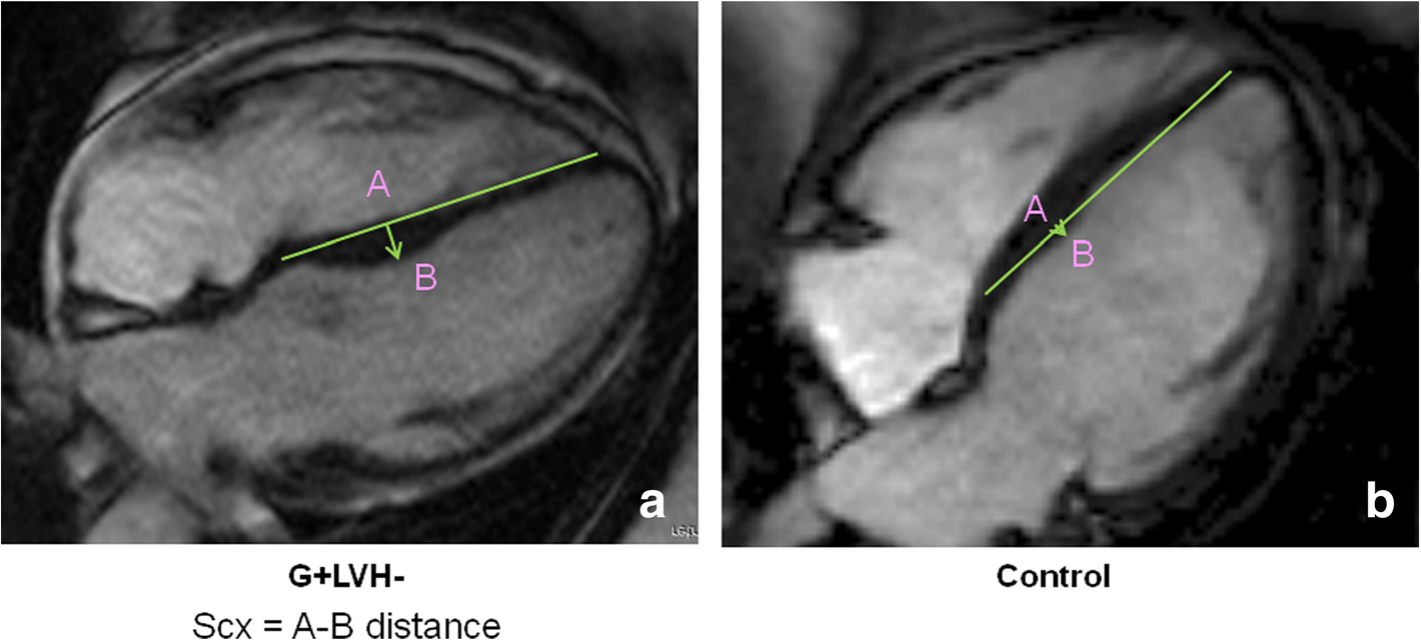
Measurement of septal convexity (SCx) in apical 4 chamber view: performed as the maximal distance (A to B) between the LV endocardial border (B) and the intersection point (A), perpendicularly to a reference line joining at mid-wall the level of tricuspid valve insertion (C) and the apical right ventricular insertion point into the LV (D) in a 49-year old G + LVH- male (**a**), and in a matched healthy control (**b**)

**Fig. 2:**
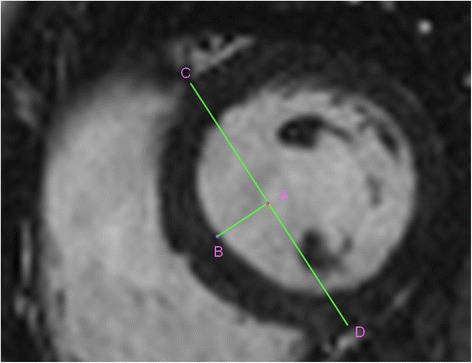
Measurement of septal convexity in short axis: views at papillary muscle level as the distance (A to B) between septal LV endocardial border (B) and the perpendicular intersection point (A) of a reference line connecting the epicardial RV insertion points into LV (anteriorly: C, and inferiorly: D)

The convexity of the interventricular septum into the LV was measured from the apical 4-chamber view as the maximal distance between LV septal endocardial border at mid LV level and a line connecting mid-wall points at the level of tricuspid valve insertion and at the level of apical right ventricular (RV) insertion on the LV (Fig. 1). Septal convexity (SCx) was expressed positively. In case of concavity (corresponding to convexity into the RV – the normal arrangement), the measure was expressed negatively. In addition, on the 3 consecutive short axis views at papillary muscle level, an evaluation of the septal convexity was performed measuring the maximal distance between LV endocardial border and a reference line joining the epicardium of the LV-RV insertion points (B to A) (Fig. 2).

### Measurement of other previously-described markers of HCM

LV mitral angle, mitral papillary muscles angle, and LV-aortic root angle were analyzed on the open source software OsiriX® (http://www.osirix-viewer.com) as previously described [20]. The angle between LV outflow tract and basal septum was also analyzed. Left ventricular end-diastolic sphericity index was measured (as previously described), as the ratio of LV end-diastolic diameter measured in short axis view at papillary muscle level (septal to lateral wall distance) divided by the LV end-diastolic long axis diameter measured in the apical 4 chamber view [12]. LV end-diastolic and end-systolic eccentricity indexes were calculated in the short-axis at the level of papillary muscles as the ratio of septal to lateral wall distance divided by inferior to anterior wall distance [11].

LV end-systolic left atrial areas were measured by planimetry on 4-chamber view. Anterior mitral valve leaflet (AMVL) length was measured using the method previously described by Maron et al. [2] Additionally, the mitral valve annulus was measured in mid diastole in the apical 3 chamber view. The 3 long-axis views and a modified 2-chamber view (transecting RV insertion points) were evaluated for the presence of myocardial crypts defined as focal myocardial defects with a depth of ≥50 % of the adjacent myocardium [5].

### Statistical analysis

Descriptive data were analyzed for normality using the Shapiro-Wilks test and were expressed as mean ± standard deviation except where otherwise stated. Categorical variables were compared using χ^2^ tests. Non-categorical data were directly compared using paired t test. An optimal threshold value for SCx within this case–control population was calculated as the Youden Index derived from the area under the receiver operating characteristics curve. Mean variability within and between readers was evaluated using the mean of differences between two measurements. Paired measurements for repeatability of SCx were evaluated using the Bland–Altman method [28]. A 2-sided p value <0.05 was considered significant. Statistical analysis was performed using SPSS for Windows version 20.0 (Chicago, IL).

## Results

### Patient characteristics

The study population consisted of 36 G + LVH- individuals (aged 15–62 years) with complete DICOM short and long axis images from within the original Heart Hospital cohort. G + LVH- cases deriving from 30 unrelated families, were compared to 36 matched healthy volunteers (aged 14–59 years) (Table 1). Children and adolescents (aged <18 years) comprised 20 % of the G + LVH- cohort. Thirty five unique mutations in a total of 6 sarcomere genes were represented across the G + LVH- population: MYBPC3, n = 16; MYH7, n = 6; MYL2, n = 0; TNNT2, n = 4; TNNI3, n = 7; and ACTC1, n = 2, with mutations in MYBPC3 (47 %) and MYH7 (17 %) being most prevalent. There was one multiple-mutation carrier (MYBPC3 and TNNI3).

**Table 1:** Demographic and imaging characteristics of G + LVH- compared to controls

Characteristics	G + LVH-	Controls	Univariate
	(n = 36)	(n = 36)	p value
Male gender, n (%)	12 (33)	12 (33)	>0.99
Age, years	31.3 ± 13.8	33.4 ± 12.2	0.053
Ethnicity*	A = 35	A = 35	>0.99
	D = 1	D = 1	0.60
Body surface area, m^2^	1.8 ± 0.2	1.8 ± 0.2	0.60
Septal wall thickness, mm	8.9 ± 1.9	8.4 ± 1.2	0.13
Posterior wall thickness, mm	6.4 ± 1.4	6.4 ± 1.4	0.86
Septal/posterior wall thickness ratio	1.41 ± 0.31	1.36 ± 0.31	0.59
LV mass index, g/m^2^	57.8 ± 11.7	59.1 ± 12.8	0.65
Relative wall thickness	0.31 ± 0.05	0.29 ± 0.04	0.039
LV end-diastolic volume index, mL/m^2^	73.2 ± 9.8	74.4 ± 11.2	0.60
LV end-systolic volume index, mL/m^2^	21.4 ± 4.4	23.7 ± 5.8	0.048
LV ejection fraction, %	70.8 ± 4.3	68.3 ± 4.4	0.022
LV long axis end-diastolic diameter, mm	92.2 ± 7.6	92.7 ± 6.3	0.63
LV transversal end-diastolic diameter, mm	47.3 ± 3.7	48.8 ± 3.9	0.10
LV end-diastolic sphericity index	0.52 ± 0.05	0.54 ± 0.05	0.21
LV end-diastolic eccentricity index	0.90 ± 0.06	0.90 ± 0.06	0.62
LV end-systolic eccentricity index	0.88 ± 0.06	0.89 ± 0.07	0.55
LV-mitral angle,°	83.2 ± 5.7	83.6 ± 3.5	0.54
Mitral papillary muscles angle,°	112.4 ± 20.0	109.3 ± 15.6	0.44
LV-aortic root angle,°	137.9 ± 7.7	137.1 ± 7.2	0.84
LV outflow tract-basal septum angle,°	141.9 ± 14.9	142.5 ± 14.7	0.82
Septal convexity (A4C) into LV (SCx), mm	5.0 ± 2.5	1.6 ± 2.4	<0.0001
Septal convexity Sax1 at PM level	10.7 ± 3.7	12.0 ± 3.1	0.11
Septal convexity Sax2 at PM level	10.1 ± 3.4	11.5 ± 3.5	0.098
Septal convexity Sax3 at PM level	8.9 ± 3.3	9.8 ± 3.9	0.37
Myocardial crypts (≥1), n (%)	11 (30 %)	3 (8 %)	0.017
LVOT end-diastolic diameter, mm	21.8 ± 2.4	21.6 ± 1.9	0.73
LVOT end-systolic diameter, mm	18.2 ± 2.2	18.8 ± 2.0	0.21
Anterior mitral valve leaflet length, mm	23.5 ± 3.0	19.9 ± 3.1	<0.0001
Mitral valve annulus diameter, mm	29.4 ± 3.9	30.7 ± 3.2	0.18
Left atrial area index, cm^2^/m^2^	10.9 ± 1.5	10.7 ± 1.4	0.73

*Ethnic headings are defined in accordance with UK Office for National Statistics guidance on national standards: A indicates white; B, mixed; C, Asian or Asian Black

D, black or Black British; E, Chinese or other ethnic group (including Arab)

*LV* Left ventricular, *A4C* Apical 4 chamber view, *PM* Papillary muscles, *SAX* Short axis views at 10 mm interval at PMs level (1,2,3), *LVOT* Left ventricular outflow tract

### Morphological findings: septal convexity and other measurements

**Fig. 3:**
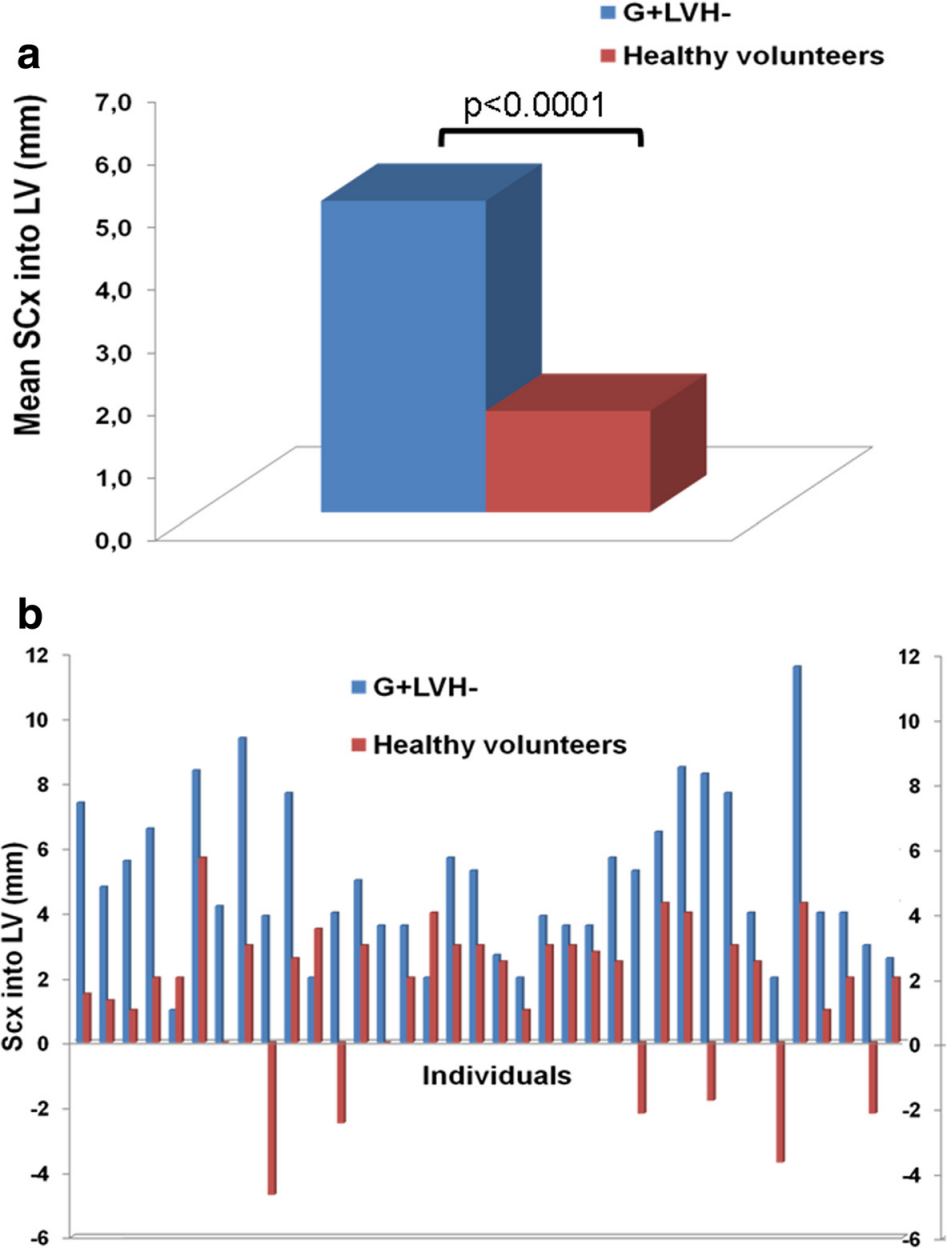
**a** Mean SCx in G + LVH- compared to healthy controls. **b** SCx for each G + LVH- and corresponding matched healthy control

Baseline CMR parameters (Table 1) for G + LVH- patients were similar to the control group, except for LVEF, LV end-systolic volume index, and AMVL length. There were two other differences: septal convexity into LV (5.0 ± 2.5 *vs*. 1.6 ± 2.4 mm, p < 0.0001, see Figs. 3a and b) and relative wall thickness (0.31 ± 0.05 *vs*. 0.29 ± 0.04 mm; p = 0.039) were greater in G + LVH- subjects than controls (Table 1). Convexity of the septum evaluated in short axis was not significantly different compared to healthy controls (Table 1). There were no significant differences between G + LVH- and control group concerning LV outflow tract diameter, the different angles, LV sphericity, and eccentricity indexes. Figure 1 depicts an example of convexity of the septum into the LV in a G + LVH- subject compared to the matched healthy control.

**Fig. 4:**
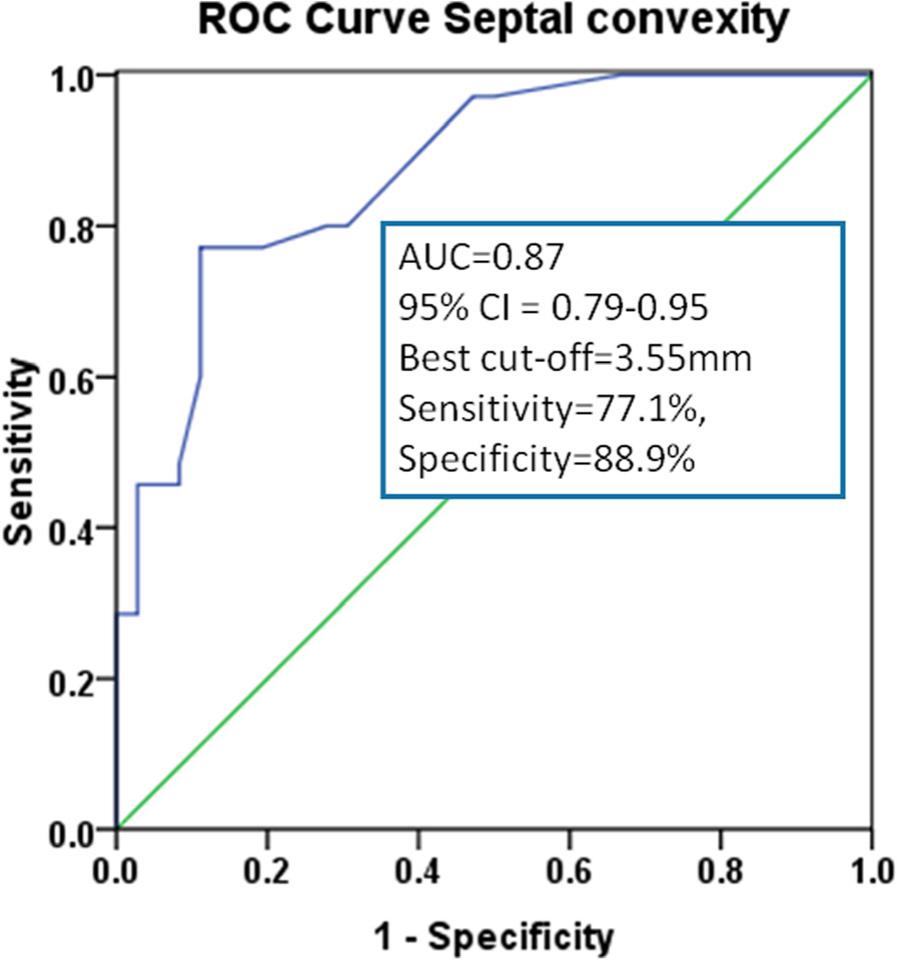
ROC curves for the SCx. SCx ≥ 3.55 mm had optimal sensitivity and specificity to differentiate the two groups

**Fig. 5:**
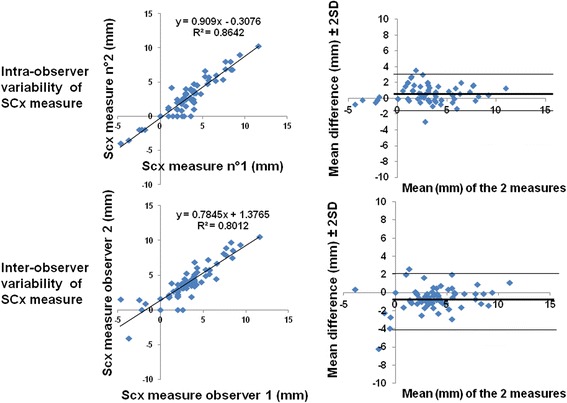
Bland-Altman analysis: intra- and interobserver variability of SCx measurements

Figure 4 displays the receiver operating characteristics curve analysis of LV septal convexity into LV to predict mutation carriers. AUC was 0.87 (95 % CI = 0.79−0.95). The cut-off point providing both the best balance between sensitivity (77 %) and specificity (89 %) was of >3.55 mm. Figure 5 depicts the good intra- and inter-observer reproducibility for SCx measurement according to Bland-Altman analysis.

As expected, myocardial crypts were more common in G + LVH- patients (11/36; 30 vs healthy volunteers 3/36; 8 %; p = 0.017). G + LVH- patients with at least one crypt had mean SCx values of 6.0 ± 3.1 mm while patients without crypt (n = 25) had a mean value of 4.6 ± 2.2 mm (p = 0.12).

G + LVH- individuals with isolated MYBPC3 mutation (n = 16) had a mean SCx of 5.3 ± 2.7 mm while individuals with other mutations a mean SCx of 4.7 ± 2.3 mm (p = 0.45). Individuals with thick filament mutations (MYBPC3 and MYH7) tended to have greater SCx than individuals with thin filament mutations (TNNT2, TNNI3, and ACTC) (5.4 ± 2.5 mm *vs*. 4.2 ± 2.4, p = 0.16).

Late gadolinium enhancement was absent in all G + LVH− patients and healthy volunteers.

## Discussion

These data show that in subclinical HCM, gene mutation carriers with normal ECGs and no LVH (G + LVH-) exhibit abnormal convexity of the interventricular septum into the LV. Four other features described here (crypts, AMVL length, and markers of elevated LV systolic function and apical abnormal trabeculae) have been previously identified using CMR [2, 7], as well as myocardial fibrosis [8, 9] and diastolic dysfunction on echocardiography [10]. These findings represent the known subclinical HCM phenotype.

Patients with HCM are at risk of developing complications such as heart failure, atrial fibrillation, stroke, and sudden cardiac death. Consequently, the detection of a subclinical HCM phenotype [29–32] in relatives of probands may facilitate closer clinical surveillance [33], and may provide markers for future therapeutic trials, particularly where genetic analysis is non-contributory [34].

This paper advances the field by the identification of one new feature – abnormal septal convexity. This was found by increasing the collaboration between CMR experts and experienced echocardiographers with the sharing of skills and ideas – the CMR approach alone previously adopted by our group missed septal convexity. The development of academic silos between imaging modalities should be resisted: breaking them down increases insights for patient care. Reverse septal curvature is well known in overt HCM patients to be related to a higher incidence of pathological gene mutation detection (79 % of the HCM patients with MYBPC3 mutation and absent in those without the mutation in one paper) [15]. More recently, Binder et al. described an important genotype-phenotype relationship linking the genetic substrate to the morphologic shape [18]. The analysis of a large cohort of genotyped and echocardiographically characterized patients reveals that nearly 80 % of HCM patients with reverse septal curvature have a positive genetic test for myofilament HCM, whereas the same genetic test is positive in fewer than 10 % of patients clinically diagnosed with HCM, but having a sigmoidal septal morphology [18]. However, it has not been previously described that reverse septal curvature could be a subclinical characteristic of HCM. This is addressed by our present study.

Septal convexity is a straightforward measure that can be taken from a standard four chamber cine image, and we provide a cut-point for analysis. Short axis views did not show the curvature – we think that part of the curvature is the centre point of the blood pool cavity not lying on a straight line – the familiar “banana shaped” cavity from overt HCM. Thus a “stack of discs” that are the short axis cines are not aligned, but any one SA view can be normal; only a view (4 chamber) showing the cavity from base to apex fully shows the distortion. Although ultimately this measure is likely to be assessed in combination with other known subclinical features of HCM (elongation of the AMVL, myocardial crypts, etc..) this technique could potentially also be applied to echocardiographic images More sophisticated methods could be used to assess septal curvature [19], and the technique may well work on echocardiographic images.

### Limitations

This is a single centre study. Preclinical HCM requires the presence of a known pathogenic mutation – early detection is really most needed where such mutations are not present. Follow-up is necessary to confirm that this abnormality precedes or predicts the subsequent development of significant LVH. Larger prospective cohorts of mutation carriers and individuals with a pretest probability of 50 % are needed, especially to determine subclinical phenotypic differences between specific gene mutations.

We did not observe significant differences between the two groups when assessment of septal convexity was performed in short axis. This is probably due to a different approach with different benchmarks, different scale of distance, and a different direction of measurement. So, we recommend using apical 4 chamber view approach to differentiate subjects. Intra- and inter-observer variabilities were good in spite of the absolute values (only a few millimeters) of our measurements. Several factors such as ethnicity, age, gender, and hypertension might impact septal convexity. In this present case control study, our population was highly selected and predominantly comprised white females aged <60 years. No subject had hypertension. We acknowledge that in clinical practice, septal convexity into LV should be used and interpreted with caution, and used only for Caucasian relatives of HCM patients, <60 years old, and without hypertension. Echocardiographically, septal curvature has been assessed in overt HCM by a method using the radius of curves matching the endocardial border [19]. Further research should address whether repeating these analyses by CMR and their eventual semi-automation provide superior insights.

## Conclusions

Septal convexity (into the LV) is an additional previously unrecognized feature of subclinical HCM. It should be assessed in combination with other known subclinical HCM features, occurring in the absence of LV hypertrophy. Further studies with follow-up are necessary to confirm that septal convexity really precedes LVH in HCM mutation careers.

## Abbreviation

ACTC1: Cardiac α-actin
AMVL: Anterior mitral valve leaflet
CMR: Cardiovascular magnetic resonance
EF: Ejection fraction
G + LVH-: Mutation gene positive without LVH
HCM: Hypertrophic cardiomyopathy
LV: Left ventricle
LVH: Left ventricular hypertrophy
MYBPC3: Myosin-binding protein C
MYH7: β-myosin heavy chain
MYL2: Myosin regulatory light chain
MYL3: Myosin essential light chain
RV: Right ventricle
SCx: Septal convexity
TPM1: Tropomyosin
TNNI3: Troponin I
TNNT2: Troponin T
